# Supplementary material for: Probing of the internal damage morphology in multilayered high-temperature superconducting wires
Source: Nat Commun. 2021 May 25;12:3110. doi: 10.1038/s41467-021-23487-0 (PMC8149865; doi:10.1038/s41467-021-23487-0)
Supplement: Supplementary file 1 — Supplementary information [file 41467_2021_23487_MOESM1_ESM.pdf]

Probing of the internal damage morphology in multilayered high-temperature superconducting wires. Supplementary Materials

You-He Zhou[1,2], Cong Liu[1,2], Lei Shen[1,2] and Xingyi Zhang[1,2,*]

[1]*Key Laboratory of Mechanics on Disaster and Environment in Western China attached to the Ministry of Education of China, Lanzhou University, Lanzhou,*

Gansu 730000, PR China

[2]*Department of Mechanics and Engineering Sciences, College of Civil Engineering and Mechanics, Lanzhou University, Lanzhou, Gansu 730000, PR China*

S1. Samples and assembly

S2.The strain-triggered flux penetration behaviors at other temperatures

S3. Magnetic field ramping results for different given strain at different temperatures

S4. Scanning electron microscopy examination results of the crack tip along the coated conductor width direction by chemical etching

[*] Corresponding author: zhangxingyi@lzu.edu.cn (X.Y.Z), Tel:+86-931-8912480 , Fax: +86-931-8914561

S5. Element analysis results of the crack tip along the coated conductor thickness direction

S6. Examination of the Luders bands in the tensile experiment at 40K.

S1. Samples and assembly

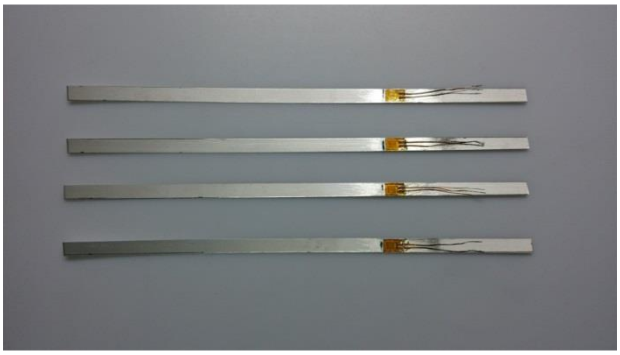

Figure S1: Testing samples with strain gage attached.

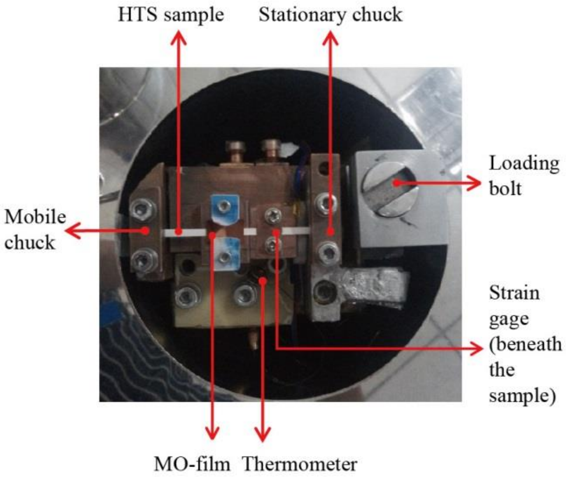

Figure S2: Photo of testing assembly.

S2. The strain-triggered flux penetration behaviors at other temperatures

The steady flux patterns of samples under different given tensile strains under zero-field-cooled (ZFC)of 64mT at 60K and 77K, 64mT are displayed in Figs. S3 and S4, respectively. In these two temperatures, both sets of flux maps can be sorted into three stages as mentioned in the main paper text. Comparing these results in three different temperatures, the sizes of regions where the local flux motions take place change with

temperatures, indicating that these places where local flux motions are still able to pin vortices and depends on temperature.

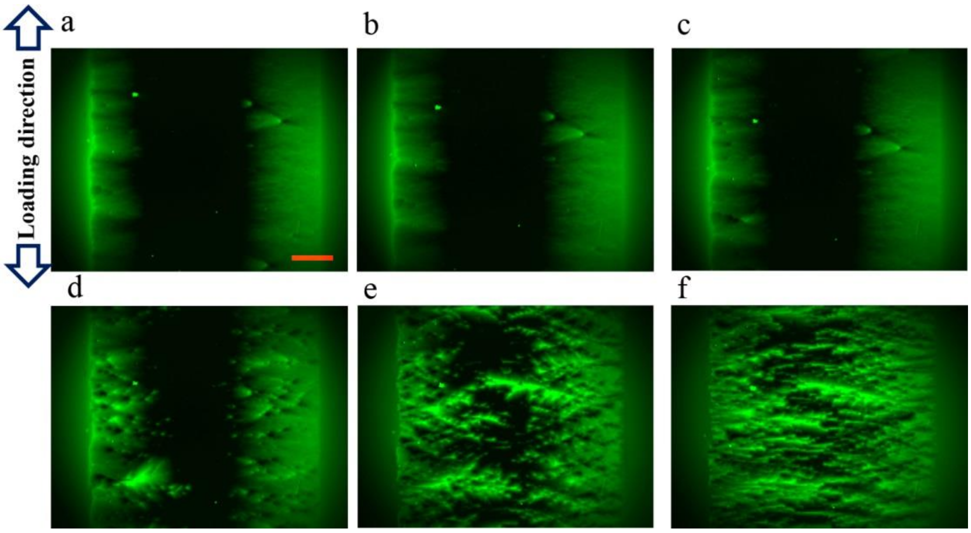

Figure S3: Flux patterns of a sample 2 with different strains at zero-field-cooled 64 mT, 60 K. Strains from **a** to **f** are 0%, 0.40%, 0.70%, 0.73%, 0.75% and 0.77%, respectively. The scale bar is 500 μm long.

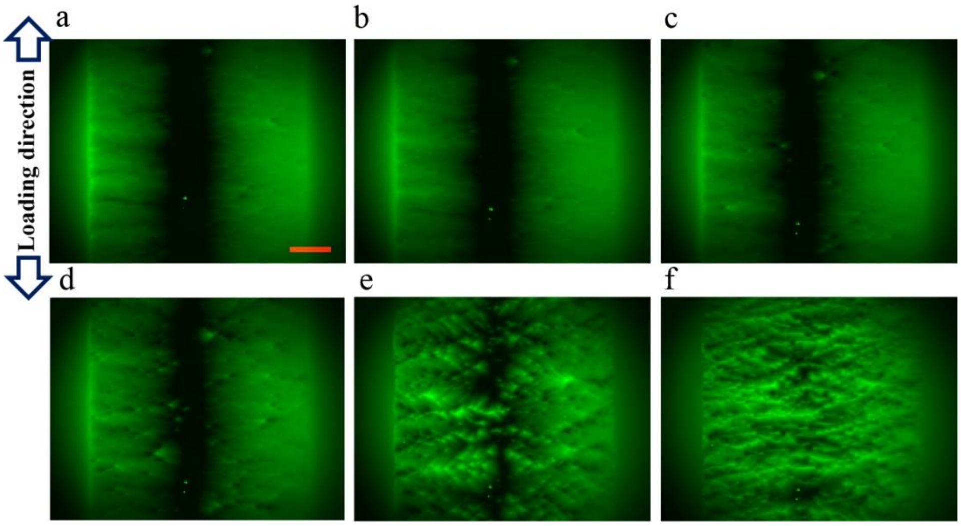

Figure S4: Flux patterns of sample 3 with different strains at zero-field-cooled 64 mT, 77 K. Strains from **a** to **f** are 0%, 0.40%, 0.68%, 0.69%, 0.71% and 0.73%, respectively. The scale bar is 500*um* long.

Sets of time variations of local flux motions of samples with ZFC of

64mT at, 60K and 77K are displayed in Figs. S5 and S6, respectively. With temperature increasing, the amplitudes of local flux motion decrease and time scales increase.

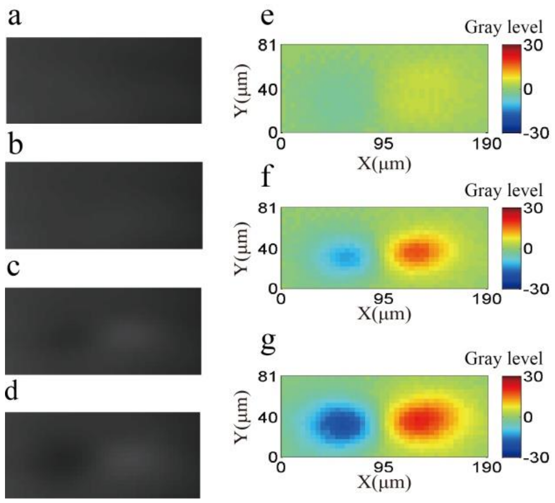

Figure S5: Time depends on the flux variation in the same local area of sample 4 strained in ero-field-cooled 40 K, 64 mT. a-d, The magnifying images of the local area at 0 ms, 20 ms, 25 ms and 60ms, respectively. **e-g**, The maps of differences of gray values between each moment of 20 ms, 25 ms and 60 ms and 0 ms.

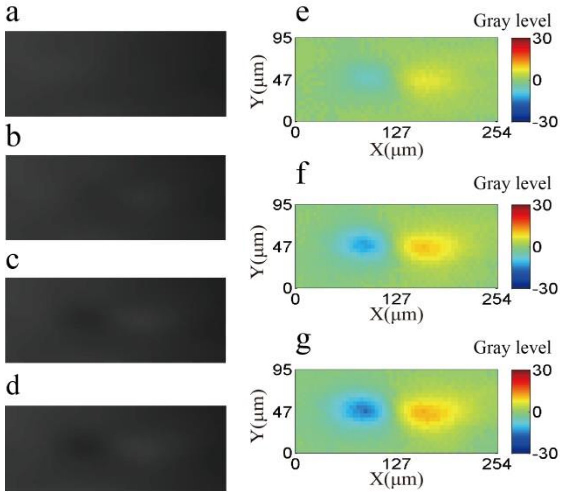

Figure S6: Time depends on the flux variation in the same local area of sample 5 strained at zero-field-cooled 40K, 64mT. a-d, The magnifying images of the local area at 0 ms, 70 ms, 90 ms and 110 ms, respectively. **e-g**, The maps of differences of gray values between each moment of 70 ms, 90 ms and 110 ms and 0 ms.

The penetration lengths of samples versus loading time with ZFC of

64mT at 60K and 77K are displayed in Figs. S7 and S8, respectively. Intermittent penetration behavior can be seen and max magnitude of speeds is no more than 1000 μm/s.

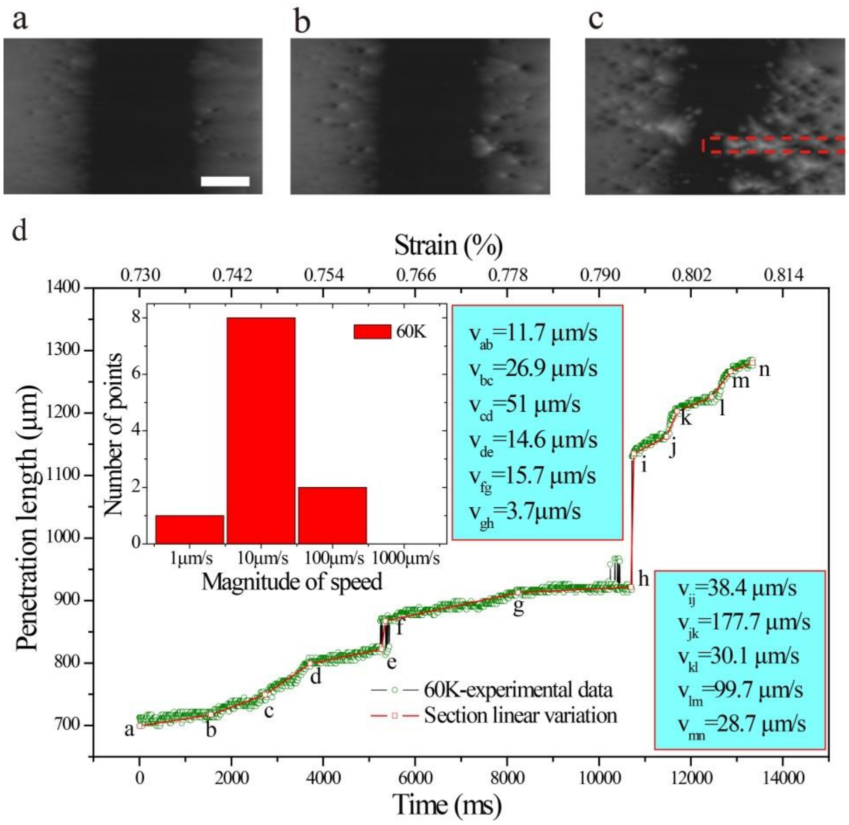

Figure S7: The flux penetration length in sample 4 at 60 K with loading time. a-b, Flux evolutions at several moments of 0 ms, 6850 ms, 13350 ms, the scale bar is 500 μm long. **d,** Penetration length in the region marked with red rectangular in **c** depends on loading time, average speeds are piecewise calculated from red curve, and their magnitude distributions are inserted in **d.**

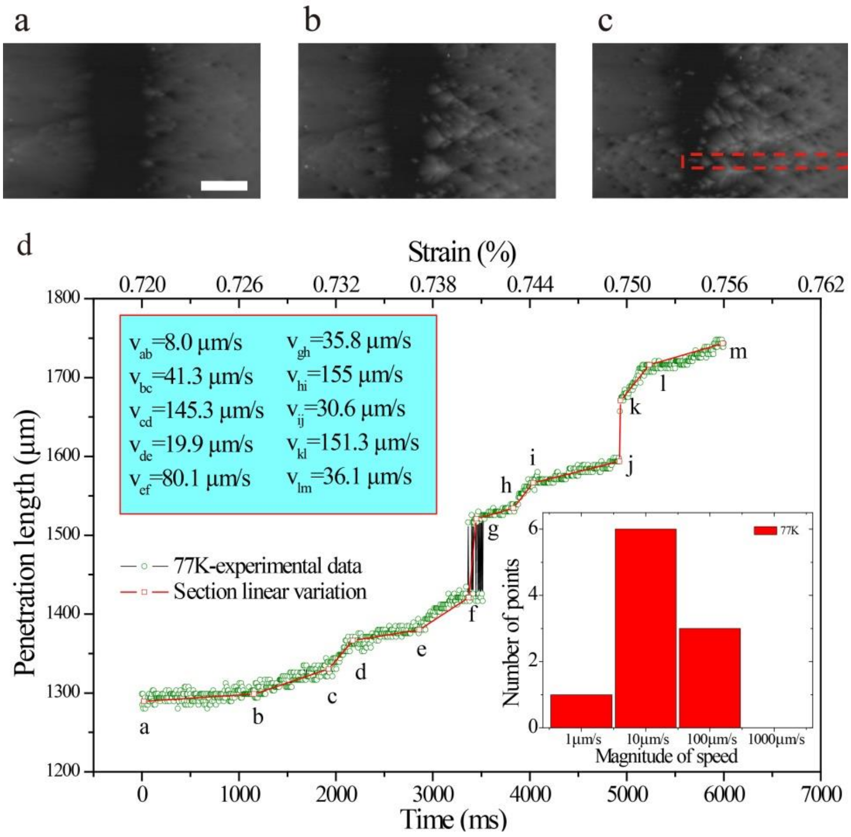

Figure S8: The flux penetration length in sample 5 at 77 K with loading time. a-b, Flux evolutions at several moments of 0 ms, 4050 ms, 6000 ms, the scale bar is 500 μm long. **d,** Penetration length in the region marked with red rectangular in **c** depends on loading time, average speeds are piecewise calculated from red curve, and their magnitude distributions are inserted in **d.**

The statistical properties in flux penetration under ZFC 64 mT at 60 K and 77 K are shown Figs S9 and S10. Differential areas in the neighboring pair of images are calculated shown in the inserted images. The normalized probabilities for the occurrence of penetrated area are plotted.

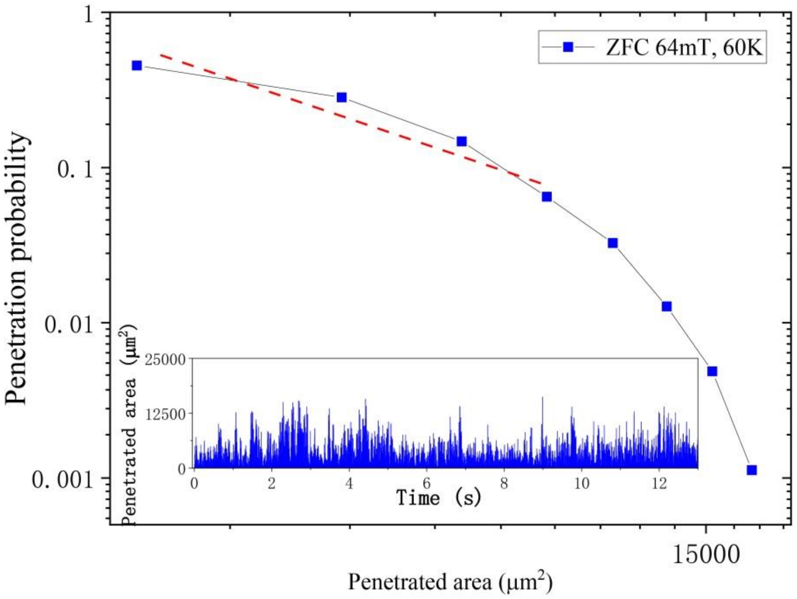

Figure S9: **Statistics of flux penetrating area (60 K).** The curve corresponds to statistics of inserted penetration area with time interval of 5 ms, the straight line with a slope of -1.33±0.31 is added as a guide for visualization.

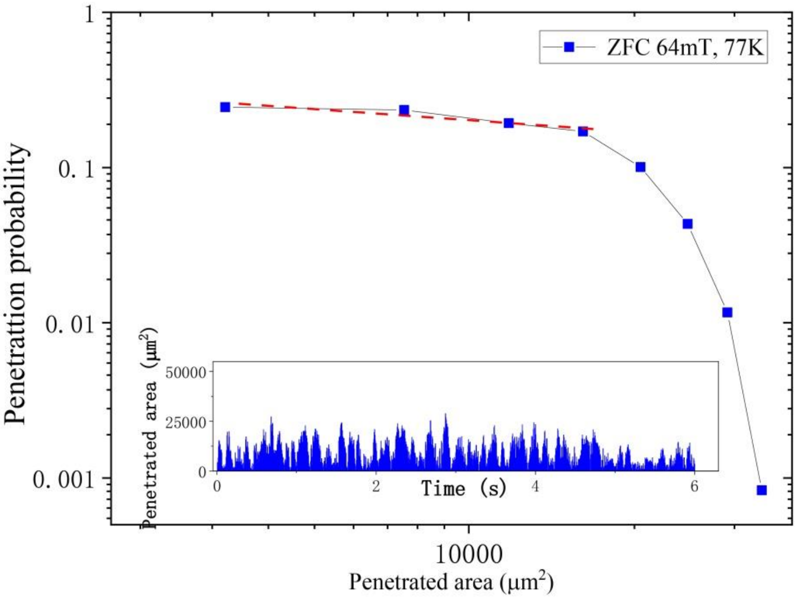

Figure S10: **Statistics of flux penetrating area (77 K).** The curve corresponds to statistics of inserted penetration area with time interval of 5ms, the straight line with a slope of -0.26±0.07 is added as a guide for visualization.

S3. Magnetic field ramping results for different given strain at different temperatures

Additional experiments are conducted as the following. First, the CC sample is zero-field-cooled (ZFC) to a targeted temperature, e.g., 40 K. In the second step, a stretching strain is applied, such as 0.60%, and an applied magnetic field is ramped up from 16 mT to 64 mT, at the same time the corresponding Magneto optical images (MOIs) are recorded. Third, both the magnetic field and the applied stretching force are unloaded and the temperature of sample is elevated to 120 K that exceeds the critical temperature of YBa2Cu3O7-δ (YBCO) for moving out the residual pinned magnetic flux. After that, the sample is re-ZFC to the targeted temperature for the next strain loading as same as in the first step. The flow chart of experimental process is shown in Fig. S11.

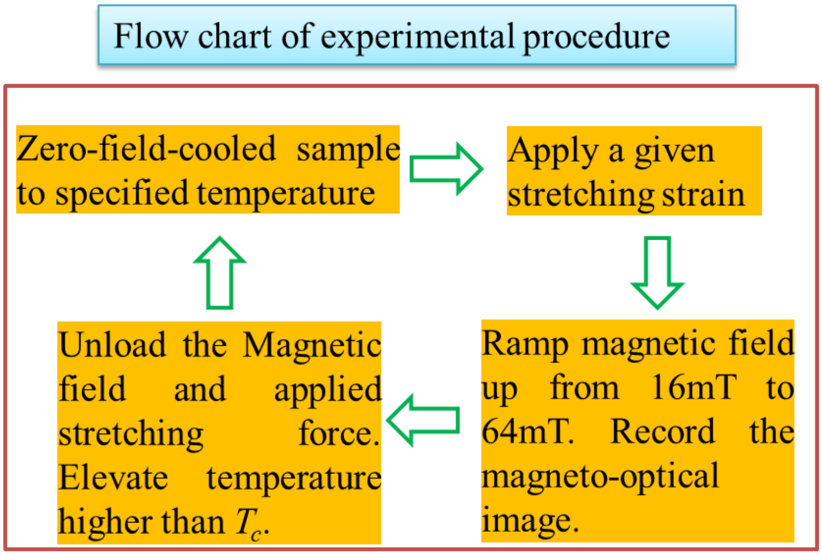

Figure S11: Flow chart of the experimental procedure.

The experiments are conducted at three different temperatures of 40

K, 60 K, and 77 K with three CC samples, e.g. 0.00%, 0.60%, 0.74% and 0.76% for 40 K as displayed in Fig. S12. In the experiment at 40 K, one can clearly find the MOI results under different applied magnetic field in strained sample with 0.60% and unstrained sample are very similar, suggesting there is no crack in the sample, and the deformation is elastic. When the applied strain is 0.74%, the local flux variation is not seen until the magnetic field is increased to a high value, this phenomenon is prominent at a higher strain value of 0.76%. Similar results can be also found in the MOI results at 60 K and 77 K, as shown in Figs. S13 and S14, respectively.

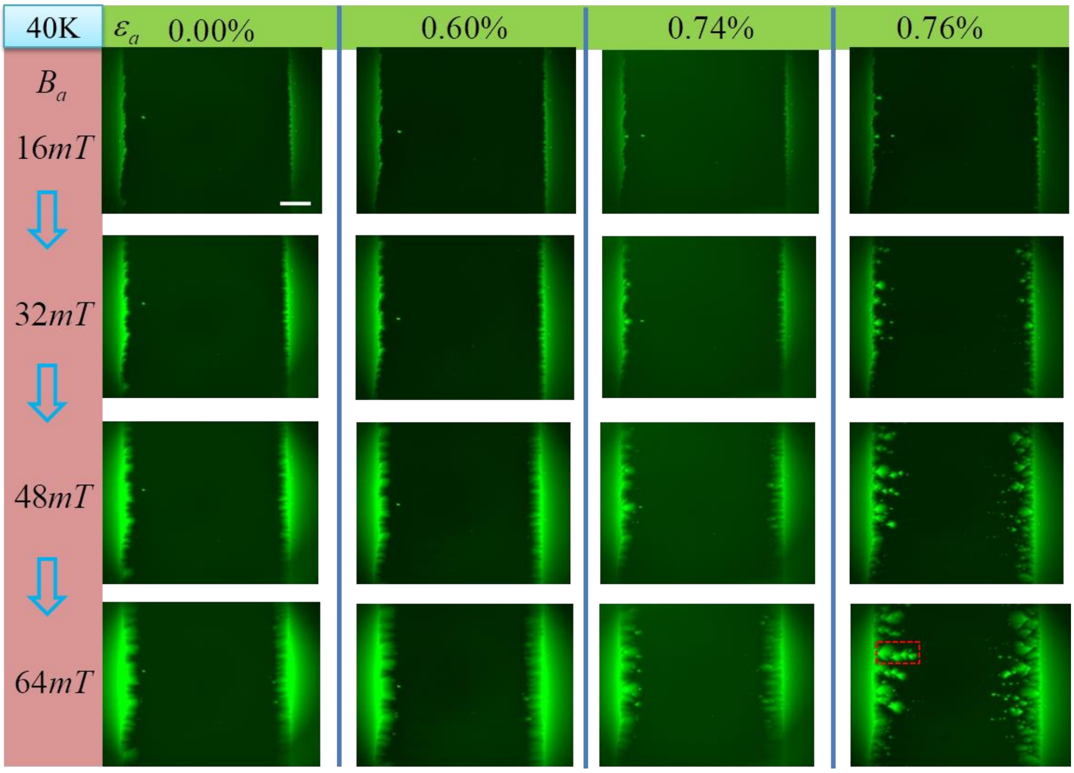

Figure S12: **Magneto-optical images (MOIs) with different applied strains at 40K**. The scale bar is 500 μm. Each column contains MOIs at a given strain under magnetic field ramped from 16 mT to 64 mT.

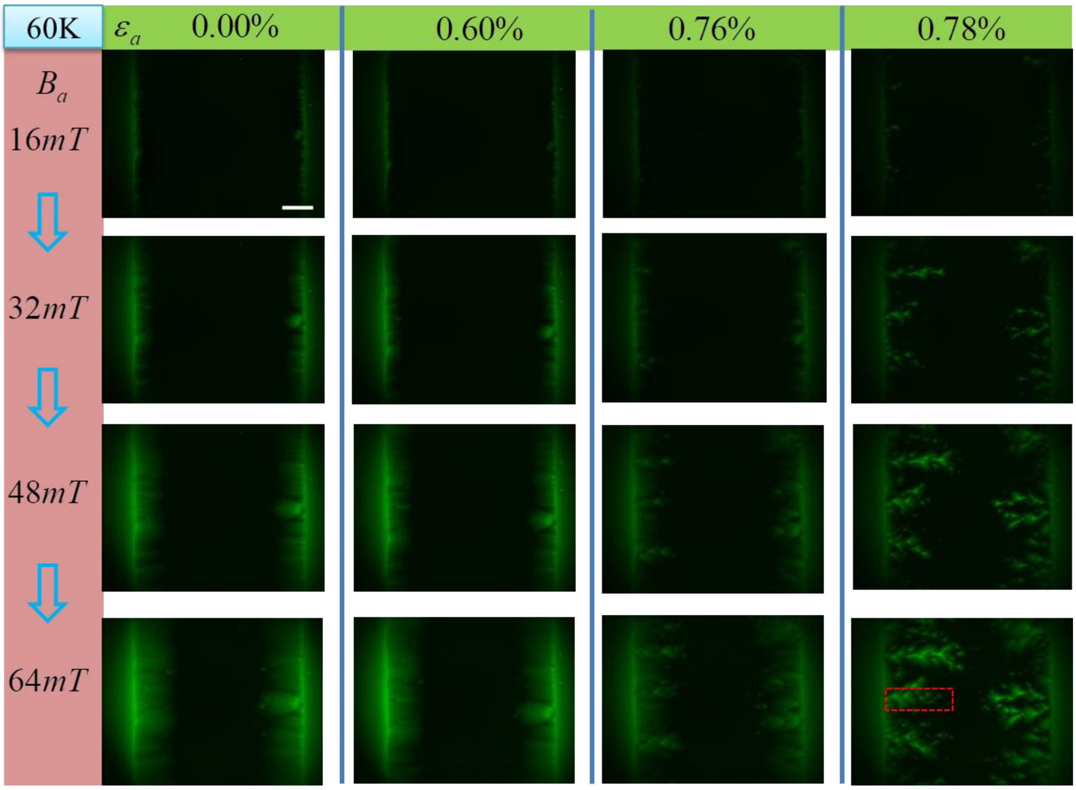

Figure S13: **Magneto-optical images (MOIs) with different applied strains at 60 K**. The scale bar is 500 μm. Each column contains MOIs at a given strain under magnetic field ramped from 16 mT to 64 mT.

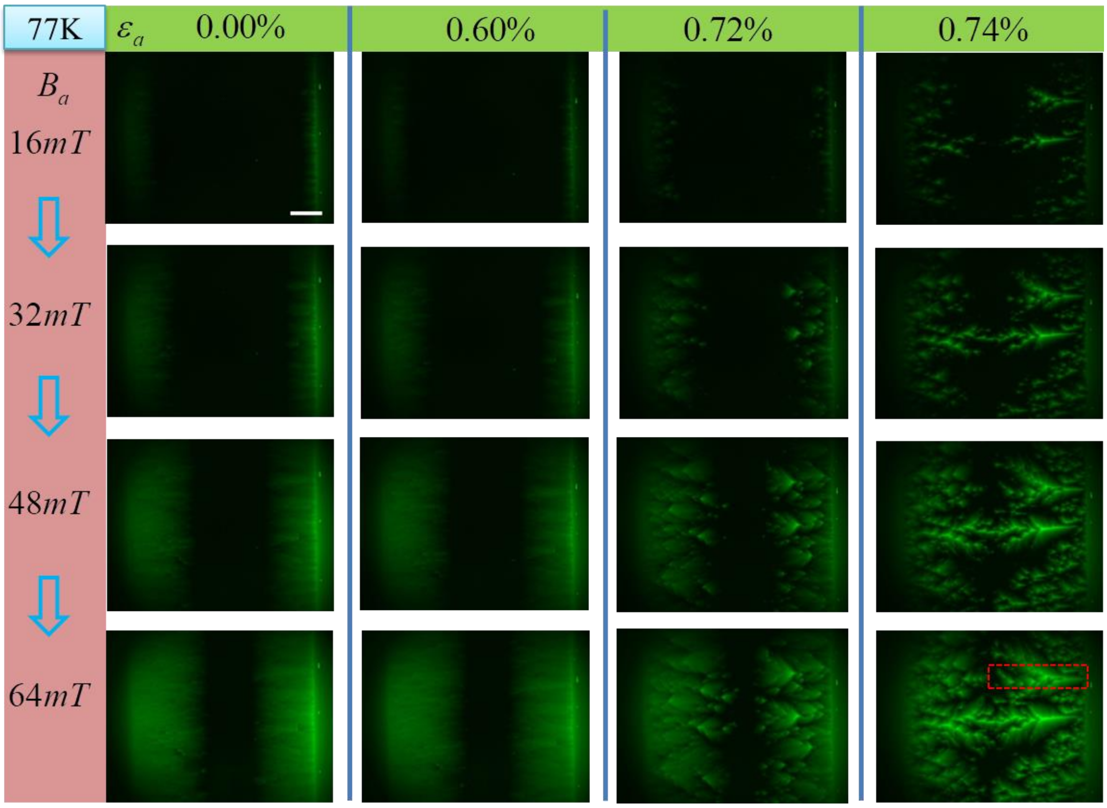

Figure S14: **MOIs with different applied strains at 77 K**. The scale bar is 500 μm. Each column contains MOIs at a given strain under magnetic field ramped from 16 mT to 64 mT.

These MOI results indicate that at low magnetic field, only a few of cracks that throughout the thickness form at the periphery of the sample. Because the totally broken part of the YBCO layer along the thickness is not able to curry any superconducting currents, resulting flux penetration in these area under low magnetic field. In contrast, the partially broken part and part without crack still have the ability of carrying superconducting current that shielding the low magnitude applied magnetic field. Nevertheless, under a higher magnetic field, due to smaller shell current density (current density multiply by unbroken thickness) of the partially broken part, a larger penetration of flux length take places compared that of that of thickness without crack, i.e. the sensibility of flux penetration against applied magnetic field of partially broken part is higher than that of unbroken part, as shown in Figs. S15**a** and S15**d**. Similar results can also be found in strained sample of 0.76% and 0.78% for 60 K, as displayed in Figs. S15**b** and S15**e**, and that of 72% and 0.74% for 77 K, see Figs. S15**c** and S15**f**.

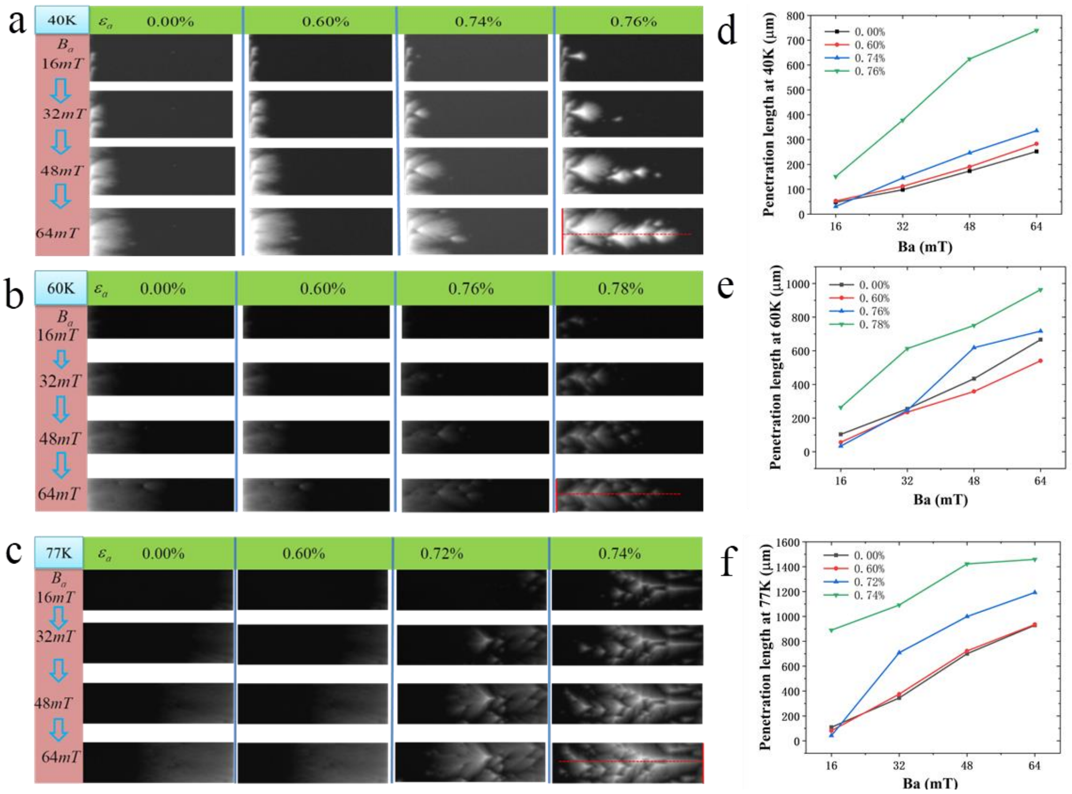

Figure S15: **In situ comparisons of local magneto-optical images (MOIs) that marked by red rectangle box in different temperature results (Figs. S12-S14). a-c** are local results at 40 K, 60 K and 77 K, respectively. **d-f** are flux penetration length results of different strain alone the red dash line at 40 K, 60 K and 77 K, respectively.

S4. Scanning electron microscopy examination results of the crack tip along the CC width direction by chemical etching.

A Scanning electron microscopy (SEM) examination on 0.75% strain-induced crack tip along the CC width direction is displayed in Fig.S16. From the in situ comparison results of Figs. S16a-S16d, it is found that with the thickness reduced by chemical etching, the crack is extended. The reason is that YBCO layer is partially broken at the crack tip, and therefore the crack below the unbroken part emerges when the unbroken part is etched. The schematic is shown in Fig 6**e**. This result has verified the indication of the MOI results.

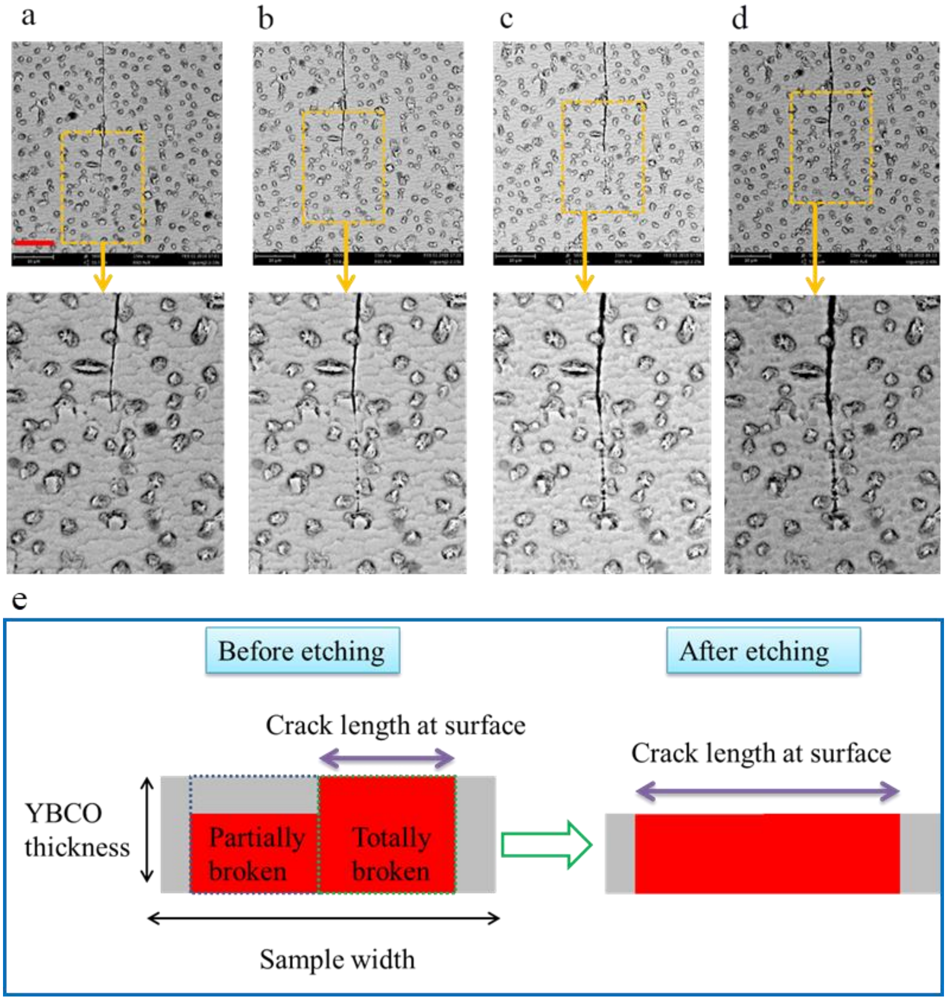

Figure S16: **Scanning electron microscopy (SEM) images of the strained sample (zero-field-cooled 40K, 64mT, strain of 0.75%)**. The red scale bar is 10 µm. **a-d** are results for etching time of 10s, 15s, 25s and 40s. **e** The schematic of the crack model before and after etching.

S5. Element analysis results of the crack tip along the coated conductor thickness direction

The elemental analysis results are shown in the supplementary Fig. S17, which manifest the crack tip existed in YBCO layer.

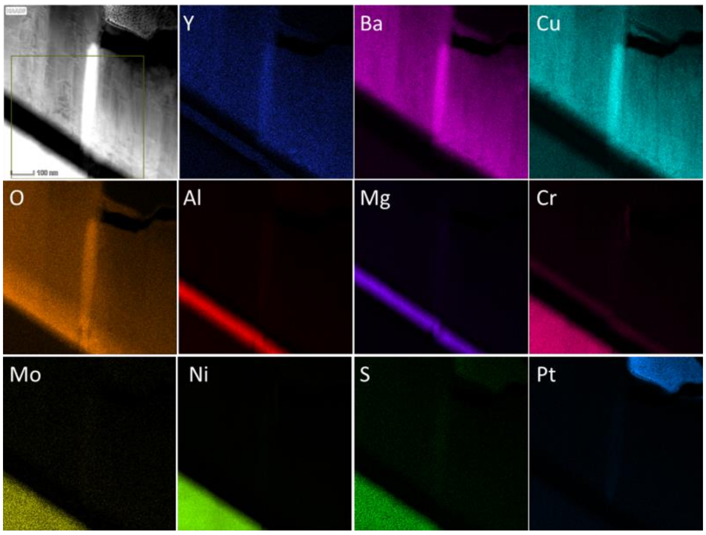

Figure S17: Element analysis at the region of the interested crack tip.

S6. Examination of the Luders band in the tensile experiment at 40 K

A tensile experiment for pure Hastelloy-C276 substrate with 4mm width is conducted at 40K with white light illumination, one can find no strip-like patterns that extended along tape width direction with nearly 45 degrees with respected to the tape length, hence no Luders band is observed at the surface as displayed in Fig. S18. The main reason is that the observation area of the magneto-optical is far away from the chuck, and the strain of the material is uniform before the crack appears, so it does not appear the Luders band.

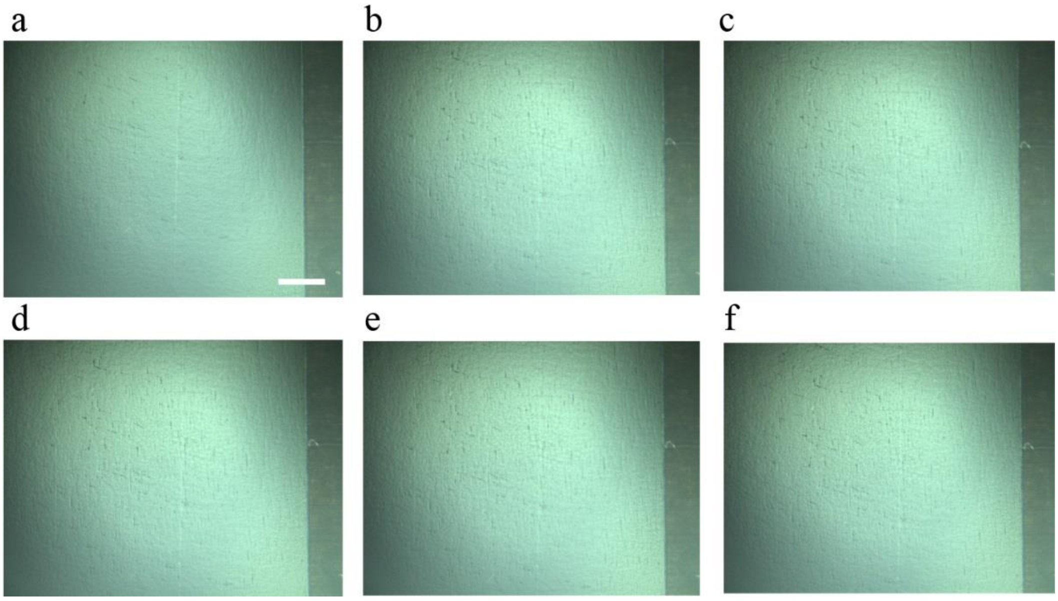

Figure S18: Images of the as received Hastelloy-C276 substrate (4 mm width) under tensile strain at 40 K. Scale bar is 500 μm. No Luders bands were found. Strains are (a) 0%, (b) 0.7%, (c) 0.8%, (d) 0.86%, (e) 0.90%, and (f) 1.0 %
